# Virtual balint group experience due to the COVID-19 pandemic

**DOI:** 10.1192/bjo.2021.367

**Published:** 2021-06-18

**Authors:** Rebecca Brown, Nicola Philips

**Affiliations:** Nottinghamshire Healthcare NHS Foundation Trust

## Abstract

**Aims:**

In the changes brought about by remote working, the local psychotherapy case discussion group (Balint Group) has developed as a remote service via video consultation. It is important to consider the effect that this change in method of delivery has had on experience.

**Method:**

An anonymous survey was distributed to determine the benefits and challenges from participants and facilitators with at least a month of virtual Balint Group experience. The open-ended survey questions captured extended answer responses from 16 students and trainees, and 5 (co-)facilitators, within Nottinghamshire Healthcare NHS Foundation Trust. The qualitative feedback was analysed by thematic analysis, identifying three main themes.

**Result:**

The first theme of practicalities was centred around access to the group. The virtual format had benefits in terms of reducing travel and time commitment and so improving attendance. However, disadvantages were in technological issues and finding a private and safe environment, individuals often not leaving the work environment on which they were reflecting.

The second theme of communication identified how virtual methods are a less natural way of interacting (for example sequential point making), losing both immediacy of reactions and non-verbal communication. There was a loss of essential communication cues, with disjointed conversation affecting contribution.

The third theme of group dynamics had some advantages, feeling less intimidating virtually. Yet disadvantages included loss of group cohesion, with participants not building the same relationships (on arriving and leaving a group space), and trust. The more subtle emotions in the group might be missed and opinions given less openly. The facilitators needed to be more directive and experienced difficulties maintaining group engagement and managing the frame.

**Conclusion:**

The advantages of virtual format are more based on accessibility and the disadvantages more experiential. There are elements of being physically remote that lead to a disembodied experience, that might impact on capacity to reflect emotionally. This might make it more difficult to identify unconscious processes and the experience might be more cognitive. There is a risk that virtually participants will feel more alone with difficult feelings and unsupported by the group.

When mental health is being affected by social isolation due to the pandemic, having groups virtually can mimic this isolation in working life. Overall the preference remained for an in-person group.

However, it was clear that access to some form of a group was important, to contain anxiety during these unprecedented times.

